# What's the Story With Blue Steak? On the Unexpected Popularity of Blue Foods

**DOI:** 10.3389/fpsyg.2021.638703

**Published:** 2021-03-02

**Authors:** Charles Spence

**Affiliations:** Crossmodal Research Laboratory, University of Oxford, Oxford, United Kingdom

**Keywords:** blue, food coloring, digital consumption, multisensory flavor perception, gastroporn

## Abstract

Is blue food desirable or disgusting? The answer, it would seem, is both, but it really depends on the food in which the color happens to be present. It turns out that the oft-cited aversive response to blue meat may not even have been scientifically validated, despite the fact that blue food coloring is often added to discombobulate diners. In the case of drinks, however, there has been a recent growth of successful new blue product launches in everything from beer to tea, and from wine to gin, arguing that coloring food products blue is more than simply a contemporary fad. In fact, the current interest in blue food coloring builds on the color's earlier appearance in everything from blue curacao to blue-raspberry candyfloss (cotton candy), and thereafter a number of soft drinks. Over the years, the combination of blue coloring with raspberry flavoring has also appeared in everything from bubble-gum to patriotic pop rocks (popping candy in The United States). Ultimately, it is the rarity of naturally-blue foods that is likely what makes this color so special. As such, blue food coloring can both work effectively to attract the visual attention of the shopper while, at the same time, being linked to a range of different flavors (since this is one of the few color-flavor mappings that are essentially arbitrary) depending on the food format in which it happens to appear. Note also that the basic descriptor “blue” covers a wide range of hues having a range of different associations, hence eliciting different reactions (be they positive or negative). While blue was once associated with artificiality, a growing number of natural blue food colorings have come onto the market in recent years thus perhaps changing the dominant associations that many consumers may have with this most unusual of food colors.

## Introduction

There is a famous anecdote about an experiment once conducted on a group of unsuspecting diners who were served a meal of steak, chips, and peas under dim illumination. Partway through the meal, the lighting was returned to normal levels of illumination, revealing to the guests that the steak they were eating was, in fact, blue, the chips green, and the peas red. Revolted by the realization, a number of the guests were apparently immediately sick. After reading about this story as a young researcher (one who was becoming increasingly interested in the impact of food coloring on the perception of consumers), I was very pleased eventually to track down what I believed to be the original citation for this anecdote, namely an article written by Wheatley (1973). First published in the trade publication *Marketing*, the article was subsequently republished 2 years thereafter in the fragrance industry newsletter, *Dragoco Report*, published by a German perfume manufacturer (Wheatley, 1975).

In recent years, my colleagues and I, as well as many other researchers have more or less accurately reported on this study as if the events described by Wheatley had actually taken place (e.g., see Thesen et al., 2004; Zampini and Spence, 2012, p. 740; Spence and Piqueras-Fiszman, 2014; Spence, 2017a; Bruno and Pavani, 2018; p. 89). The story also appears elsewhere in the academic press on food coloring (e.g., Kostyla and Clydesdale, 1978, p. 303; Cardello, 1994, p. 267, 269; Kennedy et al., 2005; Kappes et al., 2006, p. S590; see also Blackman and Kvaska, 2011)^1^, as well as having been widely covered in the popular press. For example, Eric Schlosser, best-selling author of *Fast Food Nation* (Schlosser, 2001a), did much to popularize the blue steak story, devoting three sentences to the “experiment,” in an article that appeared in *The Atlantic* (Schlosser, 2001b). Meanwhile, other mentions in the popular press that I have come across include: Fleming (2013), Poon (2014), Wollan (2016, p. 72), Nobel (2017), and Jahnke (2018).

But what if the events described by Wheatley (1973) never actually took place? This the discomforting suggestion that has recently been raised in an intriguing article by Joel Harold Tannenbaum, writing in *Gastronomica* (Tannenbaum, 2020). In a careful historical piece of gastronomic detective work, Tannenbaum has uncovered some facts about Wheatley that suggests it is unlikely that she was the one conducted the original blue steak experiment, if experiment is even the right name for what might more rightly be called an anecdote; An anecdote, moreover, that on subsequent retelling, has taken on something of the standing of an “urban myth” (see Tannenbaum, 2020). We will return later to the question of why this story, in particular, should have resonated down through the following decades.

Given the almost half century that has passed since Wheatley's (1973) article first appeared in print, it is hard to know, in hindsight, whether it refers to an actual event/experiment, and if so, it was one that was witnessed/conducted by Wheatley herself, or whether instead she is merely retelling a story heard elsewhere. Wheatley writes that: “*All perception of color is relative in two main ways. It is relative to memory and experience and it is relative to context. An experiment was carried out to illustrate the first of these two facts. Several people were collected round a table in a special form of lighting which showed the food on the plates in front of them but not its color. After they had consumed some of the meal normal lighting was resumed and the subjects found that the steak was blue, the peas red and the chips green. Almost all were violently sick.”* Wheatley continues: “*There was no pause for rationalization, the brain instinctively told the body to reject such ‘unnatural’ food, yet it is very likely that the reaction of young children to the same experiment would be far less extreme. They would not have had time to build up the very strong color associations of adults.”* (Wheatley, 1973, p. 26, 28).

Tannenbaum (2020), in his commentary, helpfully provides a little background—Jane Wheatley (a “she” often referred to as a “he” —including by myself *mea culpa*) was apparently an editorial writer at the magazine in which the article first appeared. What is more, the text itself does not provide any statistics (nor sample size, etc.), and given her background and future career path (as a successful editor), it would seem unlikely that Wheatley herself conducted the blue steak experiment that she so famously reports. Rather, she would appear simply to have been recounting a story that she had heard, or read about, elsewhere. The main focus of Wheatley's article is to highlight the various ways in which packaging color can be used by marketers. That being said, the article also uncritically recounts the suggestion from Max Lüscher, a Professor of Psychology from Basle University (Lüscher, 1969), that homosexuals adjust their TV sets to give a magenta cast or tint! This suggestion apparently being reprinted in a British Bureau of Television Advertising booklet – *The physiology and psychology of color* (Townsend, 1969; see Wheatley, 1973, p. 28, 67).

Whether or not the blue steak story provides an accurate representation of people's response to blue meat, the more important question here concerns the consumer's relationship with blue foods more generally? Is blue a desirable or disgusting color in food? It is worth noting that there have been several hundred published studies of the impact of food color in the years since Moir's (1936) first scientific publication on the topic (see Spence et al., 2010; Spence, 2015b, for reviews)^2^. While not all have demonstrated an impact of food color on people's sensory-discriminative or hedonic responses, there are now sufficient rigorous peer-reviewed studies out there to show that coloring a food or, more frequently, a beverage (given that it is simply easier to do the latter), can result in changes in the perceived identity and/or intensity of taste/flavor (see also Vanderbilt, 2015). Changing the color of a food can also have an impact on people's hedonic responses as well. Chylinski et al. (2015) have even provided evidence to support the view that blue coloring is more associated with a crunchy (rather than creamy) texture as compared to red in a creamy yogurt with almond bits (though see Christensen, 1983). As such, there can be little doubting that the color of food affects us. What is, though, special about the blue steak story is the almost visceral aversive response that can apparently be elicited be an “off-colour” in animal protein^3^.

Here, it is important to distinguish between the role that is played by color in setting our taste/flavor expectations on the one hand, and separately (though undoubtedly connected) the effect that coloring foods has on our taste/flavor perception (see Piqueras-Fiszman and Spence, 2015; Spence, 2015b, 2019b). The assumption underpinning much of the contemporary research on color-related taste and flavor expectations is that they influence, and hence modify, the experienced taste/flavor of food and drink should the latter be given that color. However, it should be acknowledged that there may be some rare situations in which the experience on sampling a colored food is not always determined simply by the expectations that are associated with that color. One such situation might be when we have reason to doubt whether the color is real (e.g., as in the case of augmented reality and virtual reality tasting experiences; see Ueda et al., 2020; Wang et al., 2020a; Xu et al., 2021).

Given the replication crisis that has been convulsing the psychological sciences in recent years it is certainly worth carefully questioning many of the more newsworthy findings that are taken as fact in the world of pop psychology (see Della Sala, 1999). After all, a dispiriting number of social psychology findings have been questioned in recent years (Resnick, 2016). What is more, the world of food psychology has also had its own dodgy data scandal to deal with, leading to the retraction of many of the articles by former leading food research Brian Wansink (see Resnick and Belluz, 2018; Lee, 2019). And that is before we get to the various oft-cited examples of marketing interventions that famously never actually happened. For instance, just take James Vicary's claim to have induced a cinema audience to drink more Coke and buy more popcorn (by 18 and 58%, respectively) simply by subliminally flashing up the words “COCA-COLA” and “EAT POPCORN” at the start of a cinema feature. It subsequently turned out that Vicary had made the entire story up (Karremans et al., 2006; Samuel, 2010, Chapter 3)!

### On the Origins of the Blue Steak Story

Oftentimes, those interested in understanding where a particular factoid or anecdote came from create citation trees to help trace back the development of the idea (e.g., Sivak, 1996; Spence, 2015a). Unfortunately, however, the citation tree in the case of the blue steak experiment stops squarely with Wheatley (1973), since neither of the references that are mentioned in her article (namely Lüscher, 1969; Townsend, 1969) mention the blue steak experiment. In fact, the likely source of her story has recently been traced back to Cheskin's (1951) book *Colors, and what they can do* first published in 1951^4^. There, Cheskin, a famous marketer in the middle decades of the twentieth century (Samuel, 2010) describes a meal with a group of people that matches pretty-closely to Wheatley's account. The same story, note, also appearing in several of Cheskin's subsequent books (e.g., see Cheskin, 1957, 1967, 1972). Given the popularity, and widespread dissemination, of Cheskin's writings (Samuel, 2010), and the similarity to Wheatley's description, this would seem to be the most plausible source for the latter's description.

Writing half a century ago, Watson (1971, p. 66–67) argued that: “*We have a deep-seated dislike of blue foods. Take a trip through a supermarket and see how many blue ones you can find. They are rare in nature and equally rare in our artificial hunting grounds. No sweet manufacturer ever successfully marketed a blue confection, and no blue soft drink or ice cream appeared on sale for very long*.” Watson was presumably not much of a fan of fairground treats, otherwise he would presumably have been familiar with the popular blue-raspberry (and pink-vanilla) cotton candy that had been a common feature of the fairground since at least the early 1950s, and possibly before (see Swarns, 2014). Once again, in this case, Watson also fails to provide any empirical support for his claim. He may well simply have been parroting Cheskin's general line on the unpalatability of blue foods.

In another anecdote about blue food, this time reported by Tysoe (1985, p. 13): “*Blue food, for instance, is regarded as bizarre and unnatural. Color experts Tom Porter and Byron Mikellides, of Oxford Polytechnic's department of architecture, report that “a group of young children taking part in a test with dyed vegetables became decidedly ill after eating harmless, blue-colored potatoes.””* However, whether or not the experiment described by Wheatley (1973) actually took place, and whether or not we choose to describe it as an “experiment,” anecdote, or merely an urban myth, the more fundamental question is whether the general claim that coloring food blue is off-putting is correct or not.

### Blue Food: Desirable or Disgusting?

There is, however, no simple answer to the latter question. This is because there is no unique meaning associated with color in food (and blue is presumably no exception in this regard). It really is all a matter of the form or substrate in which that food coloring appears. One can, I suppose, think of this as a version of Elliot's “color-in-context theory” (e.g., Elliott, 2019; see also Fechner's Aesthetic Association Principle from 1866, and recently translated into English by Ortlieb et al., 2020). Consider here only how redness in some fruits – think strawberries is associated with sweetness, whereas in the chile fruit it may be associated (rightly or wrongly) with spiciness instead (see Spence, 2018a)^5^. In both cases, redness is associated with ripeness (see Foroni et al., 2016), but how that ripeness expresses itself (as spicy or sweet) differs markedly between fruits.

Of course, as well as any literal crossmodal associations between colors and flavors (presumably based on associative learning) which may guide our flavor expectations, it is also worth noting that the visual appearance of food can take on a more symbolic meaning. This is perhaps especially clear in the case of the achromatic colors white and black, linked to purity/cleanliness and mourning, respectively (e.g., see Huysmans, 1884/1926; Weineck, 2006; Carter, 2011; Harris, 2011; Spence and Piqueras-Fiszman, 2014; Piepenbring, 2016; Spence, 2018d, 2020a; Strand, 2020). Furthermore, there is also a growing literature on the existence of more abstract crossmodal correspondences between color patches, or combinations of colors, and basic tastes and aromas (e.g., see Wan et al., 2014b; Spence et al., 2015; Woods and Spence, 2016; Woods et al., 2016; Spence, 2020b). In the case of abstract colors, it is not always altogether clear whether they are associated with tastes and flavors because of the colors of the source foods themselves, the packaging, brand color (think Coca-Cola red)^6^, or may have a more emotional (see Spence, 2019b), or symbolic connotation instead.

### Was Blue Always an Unappealing Food Color?

Blue can undoubtedly set expectations regarding the likely taste, but does this color actually impact consumption/elicit an aversive response? F. T. Marinetti famously colored white wine blue, orange juice red, and milk green in the early decades of the twentieth century (Marinetti, 1932/2014; see also Anonymous, n.d.). While the motivation remains rather opaque, given the Futurists' general mindset, one might assume that the idea was to discombobulate people simply by miscoloring drinks rather than any specific association with blue. Meanwhile, according to Tannenbaum (2020, p. 32): “*During World War II, the American horror novelist Shirley Jackson served meals consisting of blue steaks and red potatoes to baffled dinner guests at her home in Bennington, Vermont (Oppenheimer*, *1988* *: 108).”* Blue mashed potatoes may also have been served to children in the UK (as part of patriotic red, white, and blue dishes, mirroring the colors in the flag) to celebrate the end of the Second World War (though, thus far, I have been unable to track down any documentation to back up this particular claim). Britain's first celebrity chef, Fanny Cradock, was also fond of presenting her mashed potatoes in vivid colors such as purple and blue on her TV shows from the 1950s onwards (Ellis, 2007). Not everyone was a fan, though. Chris McManus of Bedford College, London is quoted in Tysoe (1985, p. 13) as saying: “*What we like for mashed potatoes is very different from what we like for clothes to wear. What would you make of green mashed potatoes or green meat? It means it's off.”*

There have also been a number of documented (albeit anecdotal) examples of famous individuals intentionally coloring foods blue so as to deliberately disconcert their dinner guests (e.g., Hitchcock and Gottlieb, 2003). For instance, Hitchcock reported how he used to use blue food dye to taint the food when hosting dinners at London's Trocadero back in the 1960s. As the famous director put it: “*And all the food I had made up was blue! Even when you broke your roll. It looked like a brown roll but when you broke it open it was blue. Blue soup, thick blue soup. Blue trout. Blue chicken. Blue ice cream.”* (Hitchcock and Gottlieb, 2003, p. 76). And in 1964, Hitchcock invited Cary Grant, his wife Dyan Cannon, and some other guests to his Bel Air home for a Christmas party. The evening started with Windex-blue martinis. Cannon (2011) writes:

“Two butlers brought large, covered plates to the table. Hitch gave them a nod, and they removed the covers to reveal slabs of prime rib. The beer smelled wonderful, but it looked awful. It was blue. Bright, turquoise blue. Then along came the side dishes: blue broccoli, blue potatoes, blue rolls ‘Do you think it's safe to eat?’ I whispered to Cary. ‘The color may be off-putting, but I'm sure it's perfectly fine,’ Cary said sanguinely. He was wrong. By the time the night was over, the two of us had worn a groove in the carpet between the bed and the bathroom.”

I certainly know from my own experience how unappealing blue foods can be, after having been one of the guests at a particularly memorable conference dinner (and how often does one say that?) at the *Art and the Senses* meeting held here in Oxford in August, 2006. We were served a blue soup accompanied by the sound of Miles Davies Blue (Spence et al., 2011, p. 208). A few years later, together with top Spanish chef Maria Jose San Román, we served pizza smothered in blue tomato sauce to members of the audience at a Spanish gastronomy conference that was about as popular as one might expect – i.e., not at all (see San Román and Spence, 2009). However, beyond these anecdotal examples, what do the scientific studies of the consequences of miscoloring foods blue show?

In one early study, Christensen (1983) presented participants (*N* = 29) with a soy analog bacon strip, an American-style cheese, as well as three other foods (margarine, orange juice, and gelatine), that they had to rate in terms of flavor intensity/quality, aroma intensity/quality, and texture. The normally-colored bacon was perceived as having a more intense flavor than when abnormally colored bright blue instead. There was, however, no impact of blue food coloring on aroma intensity for either the bacon or cheese. That said, cooking and browning the bacon apparently reduced the color difference substantially. In a conference poster, Sakai (2011) has also reported the lowering in appetizing ratings of sushi that was colored blue, something that I have also seen with my own eyes in a demonstration that I was involved in for a TV show in 2013 (see Nobel, 2017).

A more recent Japanese study reported that coloring a soup blue was unappealing to female participants (Suzuki et al., 2017). In particular, it was shown to lead to decreased ratings of palatability and appetite when compared to a normally-colored white or yellow soup. Meanwhile, Schlintl and Schienle (2020) recently published a study in which female participants were presented with images of an array of sweet foods, including chocolate-chip cookies, cupcakes, and cream cake displayed in either their normal color or else digitally colored blue, red, or black and white. Half of the participants were informed that red color in food was supposed to increase appetite, while blue color in food was supposed to suppress it. Both groups of participants then viewed the food images in the different colors and rated how much they would like to eat the food. Those foods that were colored blue (but also those that were colored red) were rated as less appealing than the original food images. However, there was no significant effect of the placebo manipulation in this study. The examples cited thus far should be sufficient to make clear that blue is sometimes an off-putting color in food however, it need not be. It really depends on the food format in which it appears, as we will see below.

It is perhaps also worth noting how all of the anecdotes and studies reviewed in this section, involved participants who saw the blue food coloring prior to their consuming/evaluating the foods concerned. By contrast, one unique feature of the blue steak story is how the true color of the food was only revealed part-way through the meal (Wheatley, 1973). I am unaware of any researchers having repeated this temporal manipulation specifically with blue foods, though related research suggests that relevant information/experimental manipulations that happen to be presented after (as opposed to before, or concurrently) with a tasting experience generally tend to have less of an impact on taste/flavor perception (e.g., Lee et al., 2006; Shankar M. et al., 2010; Wang et al., 2020b).

### Where Did the Blue-Raspberry Association Originate?

Raspberry-flavored blue candyfloss (cotton candy) has been a feature of the fairground for a number of decades (e.g., Spence et al., 2020). According to Park (2016), in 1958:

“The same year that the Food Additives Amendment became law, an April 7 article in a periodical called The Billboard: Outdoor Amusement Directory mentions a ‘new blue-raspberry flavor for snow cones’ promoted by a Cincinnati company called Gold Medal, which to this day sells shaved-ice Sno-Kones and popcorn machines for concession stands and snack vendors. In addition, Gold Medal was going ‘all out in pushing two new flavors for the floss (cotton candy) operator, grape-purple and blue-raspberry.’

In either 1970 or 1971, the blue raspberry ICEE took its place alongside red cherry as a signature flavor of the brand. It had an artificial raspberry flavor but was colored by FD&C Blue No. 1.”

**Figure 1:**
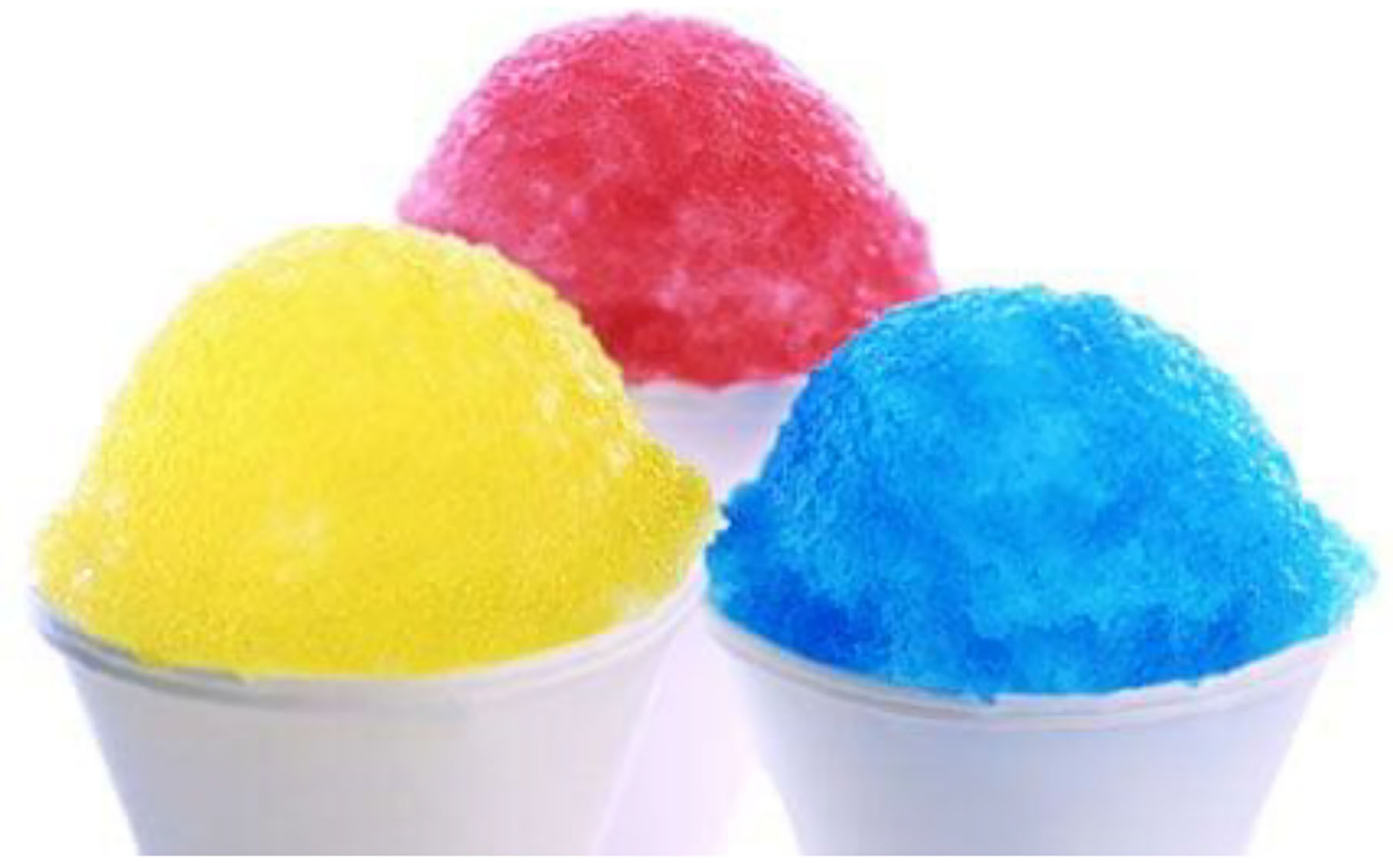
Yellow, red, and blue: how would you expect them to taste? (Photo from Kathyn Russell Studies, coutesy of Getty Images).

Hence, the blue-raspberry association would first appear to have been introduced into the marketplace in the format of candyfloss (cotton candy) at the fairground (Park, 2016; Rupp, 2016). Thereafter, the crossmodal association was likely reinforced in the minds of consumers by the widespread introduction of soft drinks such as Icee Slush Puppie, Kool-Aid, blue Jolly Rancher raspberry-flavored drink, and thereafter, the Gatorade energy drink (see Figure 1). The combination of blue coloring with raspberry flavoring also appears in Hubba Bubba gum and patriotic pop rocks (popping candy) in The United States (Greenspan, 2009; Furdyk, 2020), though pop rocks were only introduced to the marketplace in 1974. Blue tomato ketchup (along with purple and green varieties) also made an appearance in the early 2000s (see Vanderbilt, 2015).

### On the Meaning of Blue in Transparent Drinks

**Figure 2:**
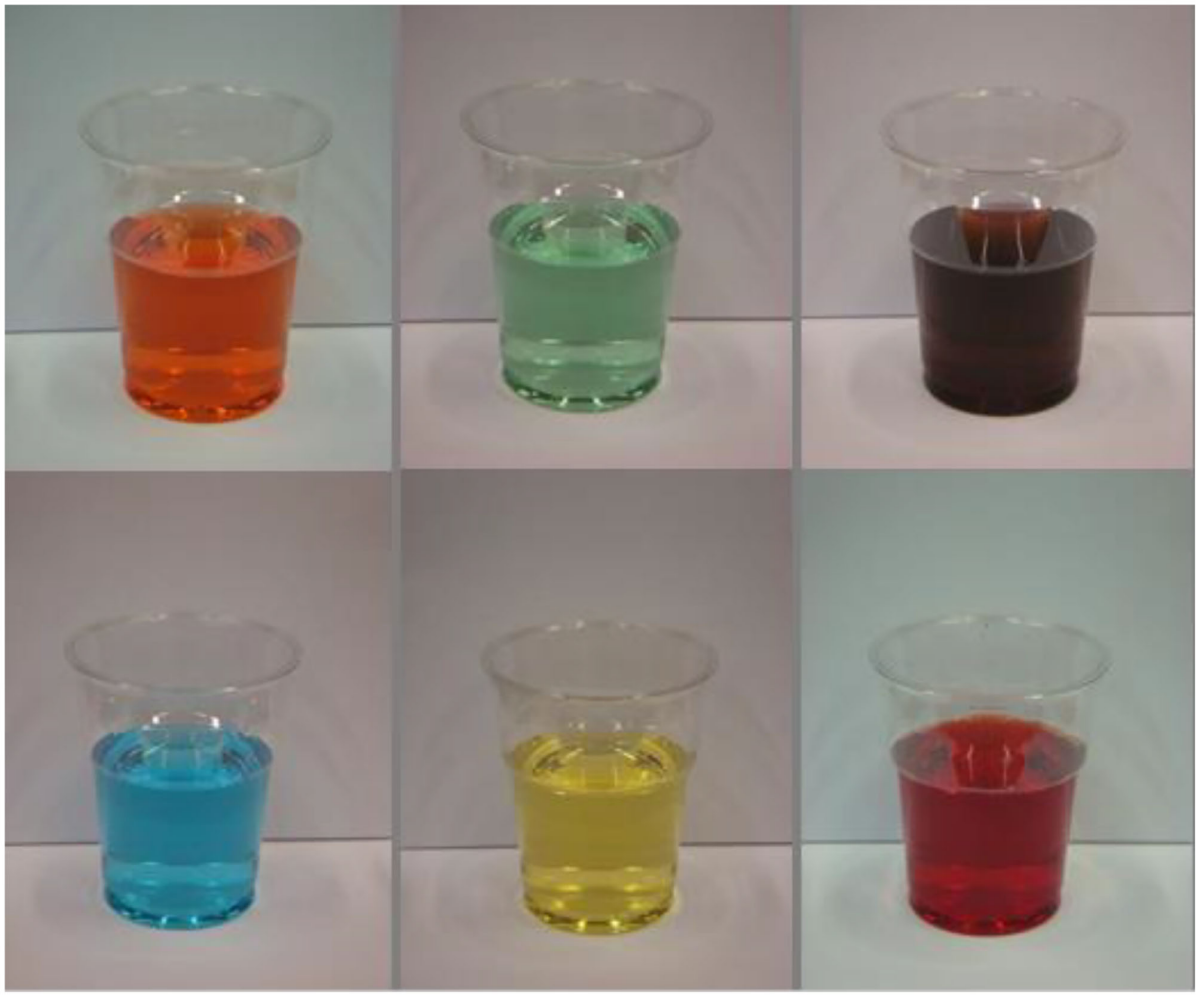
The six drinks shown to participants in the UK and Taiwan in a cross-cultural study of the flavor expectations elicited by color reported by Shankar M. U. et al. (2010).

Research conducted by my students and I a little over a decade ago, suggested that there were salient cross-cultural differences in the flavor expectations elicited by blue drinks (Shankar M. U. et al., 2010). Two groups of young adult participants, one from the UK and the other from Taiwan, were shown pictures of the same clear blue drink along with drinks of five other colors (see Figure 2). The participants were asked what flavor they expected the drinks to have. The results revealed that while the majority of the British participants expected the blue drink to taste of raspberry, the majority of the Taiwanese participants expected that it would taste of mint instead. The latter association presumably being with blue-colored mouthwash (see Parise and Spence, 2012).

**Figure 3:**
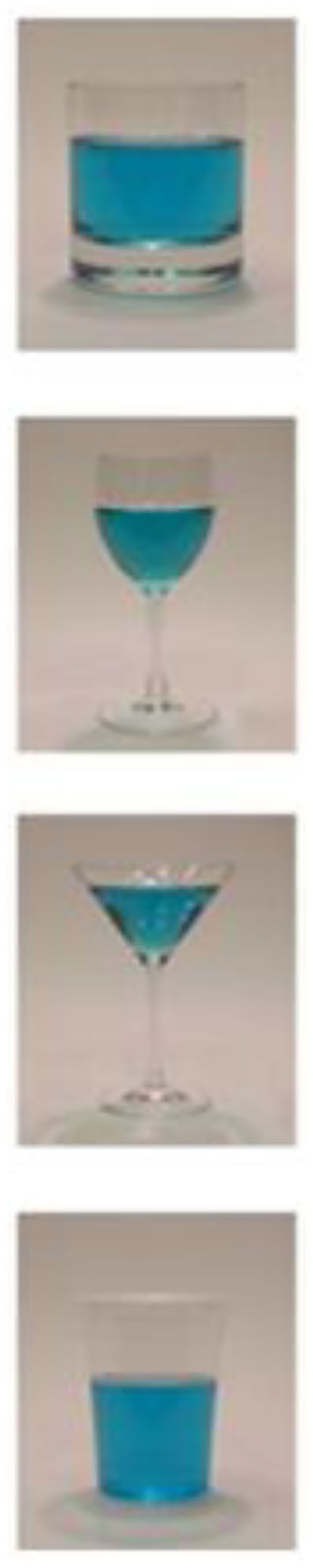
The four different glasses in which colored drinks (having one of seven colors) were presented to participants in a series of cross-cultural studies designed to assess the impact of glassware on the flavor expectations set by colored drinks in a study by Wan et al. (2014a).

Here, though, it is important to note that the interpretation of color in beverages can also be influenced by the context, specifically the glassware or drinking vessel in which it is shown. Indeed, the mint mouthwash interpretation (association) may have been primed in the Taiwanese participants in Shankar M. et al.'s (2010) study by the fact that the drinks were shown in the kind of plastic cups that one often comes across in hotel bathrooms. In our subsequent research, we have been able to demonstrate that these color-flavor expectations are modulated by the type of glass in which a drink happens to be displayed (see Wan et al., 2014a, 2015, 2016) (see Figure 3).

In 2015, as one of the experiments conducted at The Science Museum in London, and also online as part of their “Cravings” exhibition more than 5,000 people from around the world were, once again, shown drinks of six different colors and asked to pick which looked the sweetest (Velasco et al., 2016). In this case, the results revealed that the red drink garnered 41% of the votes followed, in second place, by blue (28%), and purple in third place (18%). Here, it is perhaps interesting to consider whether people expected the blue drink to be sweet because that is the gustatory association that they have with blue, or rather because the color blue is associated with raspberry flavor, and it is the latter that is considered sweet.

### On the Early Use of Blue

However, going further back in time, the first commercial blue-colored food product was probably curacao (a drink, note that also comes in orange, green, amber, and clear). The product from the Dutch island of the same name was first made in 1896, achieved international recognition when a Dutch company Bols made a blue-colored version of curacao that became internationally recognized, as the color behind many a kitsch cocktail (Martineau, 2010). According to one online source: “*Bols says they can't prove they invented the blue version, but they did start making it somewhere between 1920 and 1933.”* (Senior and Co, n.d.). According to the latter website, the color of the drink may just have been inspired by the azure color of the waters and sky surrounding this Caribbean island. Hence, in this case, the color association is, in some sense, symbolic, rather than based on a specific colored-flavored source object. The distinctive orange taste of this clear blue alcoholic drink is due to the Laraha oranges that are used to make it.

Over the last few years, various other alcoholic drinks have also been launched into the marketplace, including *Blumond*, a blue sparkling wine made from a mixture of curacao, peach and prosecco (Marchetti, 2017) and the *Gïk* sweetened blue wine brand (Hohenadel, 2016). A naturally-blue French white Chardonnay wine *Vindigo* came onto the market in 2018 (Edkins, 2018). At the same time, various blue-colored gins have also been released (e.g., the London Gin Company's Original Blue, Carter, 2011; Edgerton blue spice gin; Kiely, 2015; and the blue Magellan gin colored with iris flowers)^7^, not to mention a blue beer (Abashiri Beer's Okhotsk Blue Draft; Anonymous, 2014). Here, though, one should perhaps also be aware of the consequences of miscoloring drinks blue on people's ability to monitor their consumption of alcohol that has been documented to have, at least in the short term (Remington et al., 1997). As such, there is perhaps more ambiguity over the flavor-associations of clear blue drinks these days than perhaps there was a few decades ago. As such, the context (or glassware) in which a clear blue drink appears may be critical to constraining the flavor expectations it generates nowadays.

### On the Historical Association With Blue Coloring

Over the course of history, blue had various different associations. For instance, according to Stewart (2011, p. 58): “*The woad blue smeared by ancient Britons on their skin before battle acts as a natural antiseptic against future wounds.”* (see also Finlay, 2002). Aquamarine blue was very highly prized in the world of painting (see Stewart, 2011; see also Pastoureau, 2000). In the Medieval period, edible precious materials were desired for their eye appeal (Woolgar, 2018)^8^. In the context of food, Woolgar (2018, p. 18) writes that: “*Elite cooks were trained to produce color, and if the recipes are a sure guide, then in some environments color was highly sought after and could be an element in a great many dishes. There were recurring features. Certain dishes may usually exhibit particular colors – but there might equally be change over time, in quite a bewildering way: for example, the well-known case of ‘mawmenny’ (a minced meat, in a sauce of wine or almond milk, with spices). In the first part of the fourteenth century in England, this was colored blue; by late fourteenth century, it was commonly yellow; and by the 1420s, it was an orange-red.”* In the Medieval period, a blue color would therefore appear to be an unusual, desirable, and also temporary color in food.

Haslehurst's (1791/1814) kitchen manual provides instruction on how to create blue coloring using indigo. Rietz (1961) listed indigo, campeachy, violet, and cudbear as potential botanical sources of blue food coloring. There is, then, no simple answer to our response to blue. In the Victorian era, meanwhile, the color blue was apparently associated with poison (Carter, 2011; see also Walford, 1980; Downham and Collins, 2000; Burrows, 2009)^9^.

### Why Might Blue Be Undesirable in Food?

**Figure 4:**
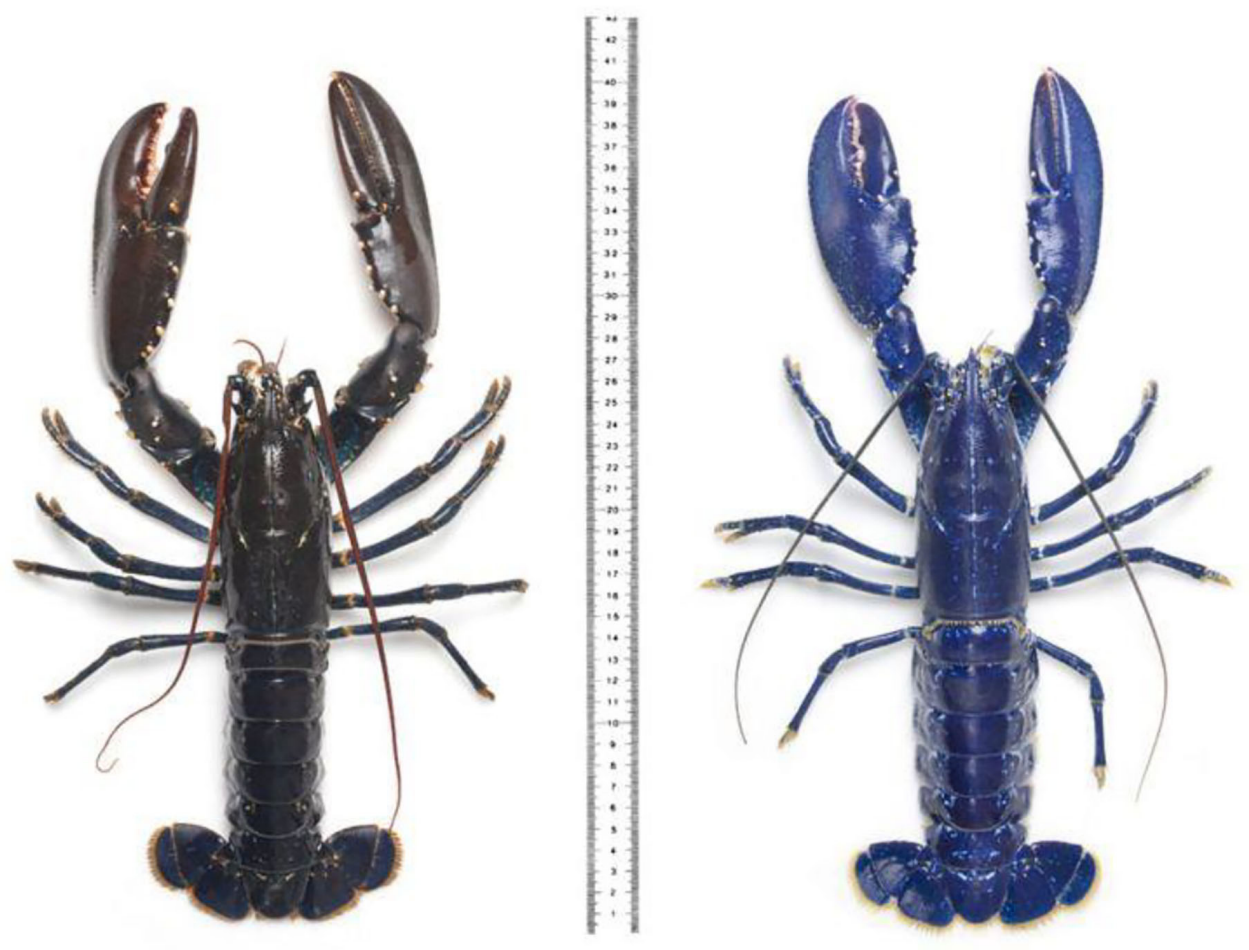
Blue lobsters are exceedingly rare. This one of the blue foods that is though to be a sign of good luck for the fishermen who catch them. There is no mention that blue lobsters are unappealing. Though note that only the inedible carapace is blue, the flesh is white. Parkinson (2016) (photo from the Science Photo Library).

Over the years, several different theories have been put forward in order here to explain why blue should be an undesirable food color. They include the suggestion that:

1. Blue is a rare appearance property of the food we consume, and this is the reason we are suspicious of it (e.g., Greenhalgh et al., 2009; Ossola, 2016). As Wollan (2016, p. 51) notes: “*Blue is a rarity among plants and animals.”* She goes on to highlight how many of the things that appear blue to us do so only because of a trick of diffraction—the scattering of light—that is the case for bird feathers, sky, ice water, and butterfly wings. Consistent with such a view, according to a recent press article, no one has yet managed to grow a blue rose (Leafe, 2019; Quest-Ritson, 2019) – *Die* blaue Blum, being a famous notion from German romantic poet and novelist Novalis (Mukhamadiarova et al., 2018).
2. Blue is a rare color in food, and therefore when we see a food that has been colored blue, our mind immediately assumes that the coloring must be artificial rather than natural (see Smithers, 2008; Fleming, 2013; Spence, 2015c). Note also that brighter colors, and this includes brighter shades of blue, also tend to be associated with artificiality (e.g., Licata, 2015). Indeed, research from Korea suggests that foods displaying higher chroma are preferred, while chroma tends to be reduced as fresh produce ages (see Lee et al., 2013). Blue No. 1 (E133 Brilliant Blue) used, for example, to achieve the blue color in curacao happens to be the only permitted food dye that crosses the blood-brain barrier (Wollan, 2016; see also Hansen et al., 1966; Kobylewski and Jacobson, 2010).
3. Blue in food looks very much like mold (see Figure 4) and we are programmed to find food that has gone off/unappealing (Piqueras-Fiszman et al., 2014; Staff Writer, 2015; see also Lee et al., 2013). According to Mühl and von Kopp (2017, p. 7): “*We instinctively recoil from food that has the ‘wrong’ color.”* while Schlintl and Schienle (2020) suggest that toxic or spoiled food often looks blue, black, or purple.

**Figure 5:**
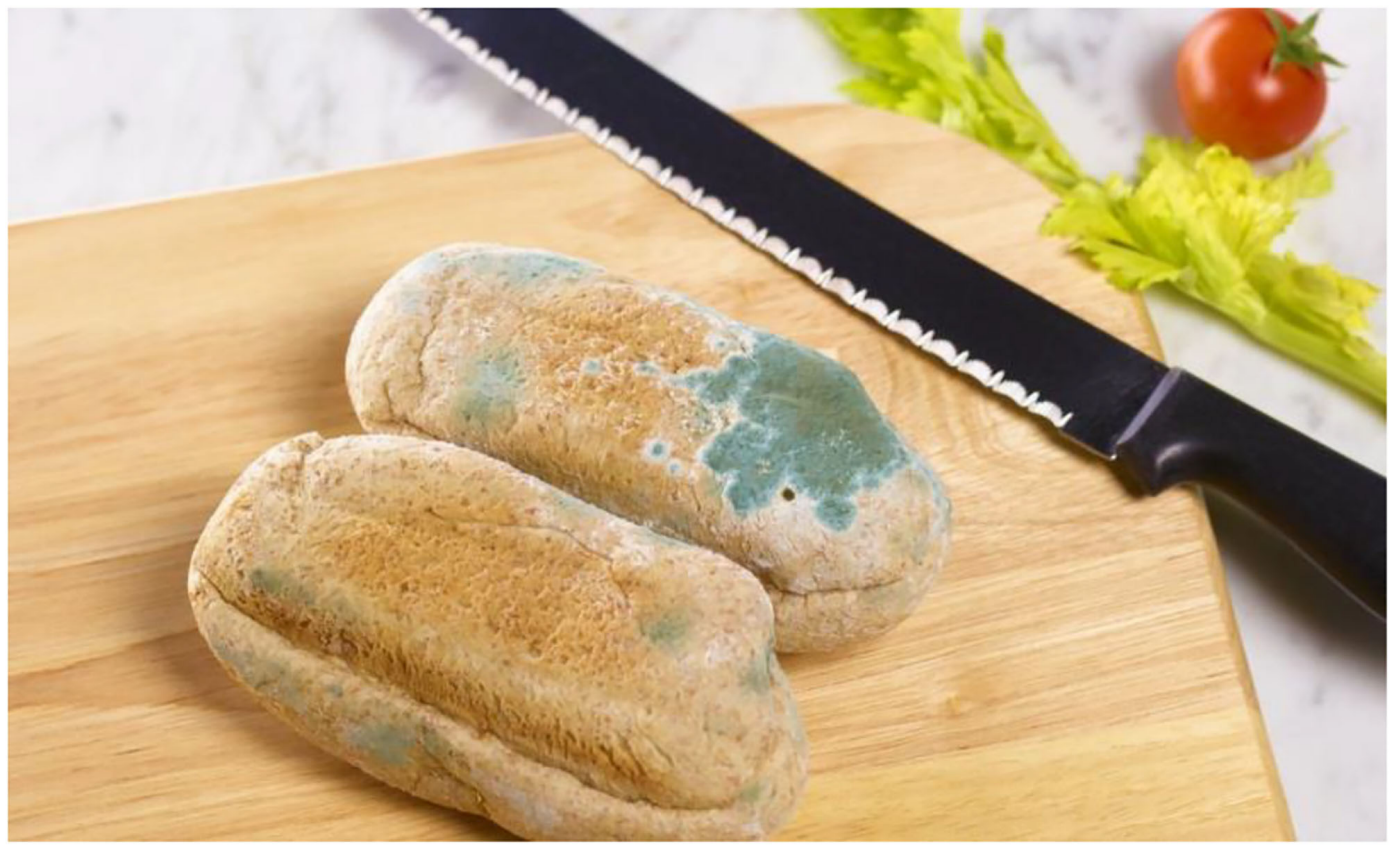
It has been suggested by some that our aversion to blue-green foods comes from the fact that moldy foods often take on this appearance (figure courtesy of Peter Dazeley/Getty Images).

There are, however, problems with all three of these accounts. While (1) may, in general be true, i.e., such foods are indeed rare, it is worth noting that rarity can actually act as a stimulant to desire – think only of truffle (see Stewart, 2011, p. 56, also links the rarity of ultramarine blue paint to the white Alba truffle in terms of their rarity). Though it is unclear whether the very rare bright blue lobsters are valued any more highly because of their distinctive color (Parkinson, 2016) (see Figure 5). Hence, rarity can either be negatively or positively valenced in the context of food.

While (2) was presumably once true it isn't obviously any more, given the rise in the number of natural blue food dyes – think pea flower, Blue Magik, iris flower, Jagua blue, etc. (e.g., Newsome et al., 2014; Brauch et al., 2016; Elgart, 2018; see Spence, 2018b, for a review). It is, though, worth noting that many of the new sources of natural blue food coloring are not without their limitations. For instance, Blue Majik can leave an undesirable taste in the mouth, reminding one commentator of bitter fishy seaweed notes (Music, 2017). Meanwhile, there is apparently an insufficient global supply of spirulina-based blue pigment to meet the needs of the food industry (Wollan, 2016). Problems with the heat and light stability of a number of the newer blue food colorings have also been reported (Jespersen et al., 2004). Indeed, the pH instability of pea flower, means that its use is limited largely to novelty foods, such as color-changing dishes, drinks, and noodles (Blake, 2017b; see also Spence, 2019a, on the range of color-changing foods that have come onto the market in recent years).

(3) Does not seem obviously true given that the blue-green moldy veining in blue cheese—think Blue Stilton, Stichelton, or Gorgonzola—are desirable attributes to many consumers. And, according to Nolan (2013), moldy foods have actually become fashionable at the higher end of modernist cuisine. For instance, the Mugaritz restaurant served a moldy apple (Adúriz et al., 2019, p. 294). That said, chefs do not appear to like using blue in the dishes they serve. This is perhaps because there is no culinary purpose for the appearance of this color other than to create eye-catching gastroporn (see Spence, 2018b). At the same time, however, the marketing and Instagrammable appeal of blue and unicorn- and rainbow-colored foods would appear to show no signs of letting-up (see Blake, 2017a,b).

### On the Recent Resurgence of Unusually-Colored Produce

While chefs rarely introduce blue coloring into the foods they serve nowadays, it would not seem inconceivable that the contemporary consumer interest in unusually-colored produce has been sparked by the molecular/modernist chefs who have been deliberately miscoloring some of the foods they serve for years (see Piqueras-Fiszman and Spence, 2012; Nolan, 2013; Spence and Youssef, 2018). Whatever the explanation, there has been something of an explosion of unusually-colored fresh produce in the supermarket aisles in recent years. UK supermarkets have started to stock everything from black garlic to orange, and yellow raspberries, and candy-striped beetroot (Anonymous, 2008; Carter, 2011). One can see the (re-)introduction of such unusually-colored produce as pine berries (white strawberries), red bananas, and purple potatoes (such as the Purple Majesty variety; Poulter, 2011), as playing to a consumer-curiosity in unusually-colored natural produce. Purple and blueish potato chips have also made an appearance in recent years (e.g., *Tyrrells* brand in UK).

The recent encouragement for consumers to eat a multi-colored diet perhaps also draws attention to the color of purple and blue foods^10^. Blue and purple fruits and vegetables have also become more desirable in recent years due to the association with higher levels of anti-oxidants (Carter, 2011). Purple majestic potatoes are higher in anti-oxidants than the white variety, as are blueberries (though, the latter are perhaps more purple when mashed). Part of the increase in the appeal of purple and blue-ish produce is, therefore, likely attributable to the healthy associations that these colors now have (see also Macrae, 2011; Poulter, 2011; De Graaf, 2016)^11^. The high levels of natural antioxidants and anthocyanins found in blue corn have also started to attract attention of researchers (Herrera-Sotero et al., 2017). As such, one might expect the consumers' response to atypically-colored foods such as blue potatoes to change as the years go by (see Paakki et al., 2016; see also Leksrisompong et al., 2012), and further support for the health benefits of blue and purple foods becomes more widely known. I would argue that the suggestion that one sometimes comes across that purple and blue are simply, and specifically unappealing colors in food (e.g., Jahnke, 2018), is simply no longer true nowadays.

It is, though, interesting to consider how the distinctive colors we associate with particular vegetables (such as white potatoes and orange carrots), could so easily have been otherwise. According to Gillian Kynoch, head of development and innovation Albert Bartlett's Purple Majesty potato variety, the reason why (in the west, at least) we tend to think of potatoes as being white lies with Sir Walter Raleigh (Carter, 2011), who brought white potatoes back to Europe, despite the fact that purple is a common color amongst traditional potato varieties in the northern part of South America he visited (Salaman, 1949)^12^. As Hisano (2019) has also noted, over the last century or so the food industry has also played an important role in standardizing the color of fresh produce – thus ensuring that we now think of bananas as yellow (not red) and oranges as always being bright orange, not green.

Carrots originated in Afghanistan and Iran with purple, red, white, and yellow varieties of this root vegetable initially all being common. According to Carter (2011), the orange variety only became popular in the 17th Century when this color was deliberately cultivated as a symbol of the House of Orange, and the struggle for Dutch Independence (cf. Banga, 1963; Dalby, 2003; Macrae, 2011; Greene, 2012, p. 81). However, according to other commentators, the popularity of orange carrots may have more to do with the fact that they do not dye the dish in which they were cooked in the way that purple carrots do (see Spence and Piqueras-Fiszman, 2014). It is somewhat ironic, therefore, to read that an ancient purple carrot variety is now finding a new role as a natural coloring (Associated Press and Cone, 2009). There are also increasingly-popular purple sweet potatoes that may have a role to play as a natural food dye too (Barclay, 2013).

Carrots of blueish nature (often called “purple,” but in fact covering wide portions of blue-ish spectrum (e.g., see Schifferstein et al., 2019, Figure 3) are taken by consumers to indicate intense taste (there may be something similar going on here with the dark and unusual colors of some varieties of heritage tomato that have started to become much more popular) and despite their current low familiarity, and hence high artificiality, are rated as much more “attractive” than white or white-green carrots, and are rated as attractive as yellow carrots.

### Blue: An Imprecise Descriptor of Food Color?

**Figure 6:**
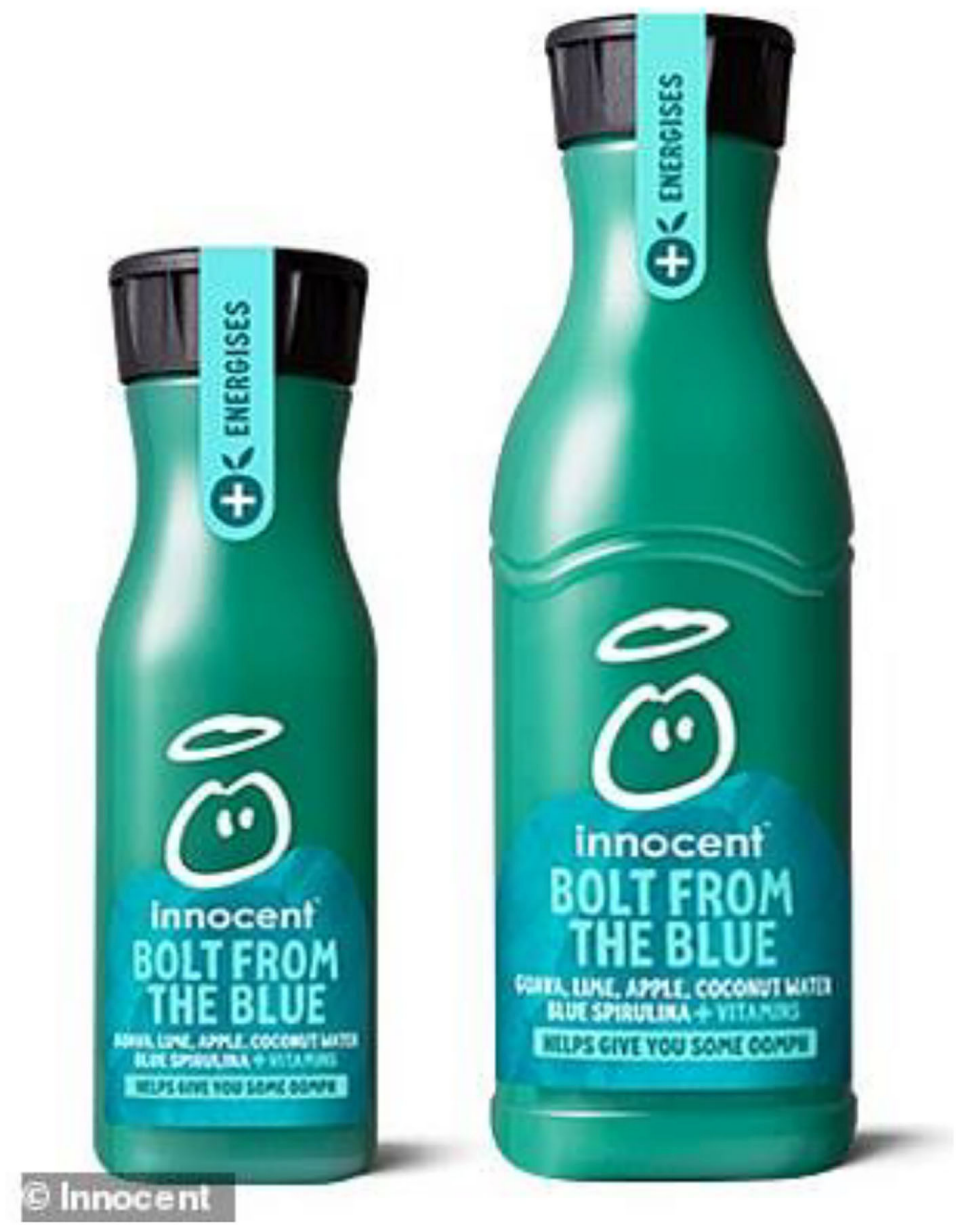
Innocent Drinks launched their Bolt from the Blue in 2018. It has been one of the companies most successful new product launches ever. Who says that blue-colored food and drink products would never sell? Though some have argued the drink is more green than blue (see Jewell, 2019) (Copyright Innocent Drinks).

Innocent Drinks' Bolt from the Blue, launched in 2018, rapidly became one of their most successful new product launches ever. The drink contains guava, lime, apple, coconut water, blue spirulina + vitamins is described as blue (see Figure 6). There has been quite some online debate about whether the drink in more green than blue (Jewell, 2019). Notice here how finding an ambiguous color for one's product can undoubtedly help generate lots of discussion, and hence free publicity, on social media. However, the more general point here is that many different hues are, rightly or wrongly, described as blue. As such, this one descriptor “blue” covers a wide range of different shades, from Oxford to Cambridge blue (cf. Schloss et al., 2011), and from the blue of so-called blue potatoes (which are more purple) to blueberries (which are perhaps more purplish when mashed). There is, of course, also the blueish-purple of a rare steak served blue/bleu (Dixon, 2013), not to mention the very artificial-looking light-white turbid blues that are seemingly favored by the food artists (Poon, 2014; Ivanova, 2015). As such, our association with blue foods likely depends on which particular shade of blue one is talking about.

One other observation relating in the ambiguous naming of blue foods comes from the case of “dark” (red-blueish) cabbage in Germany. According to a reviewer, in Northern Germany, this type (and treatment) of cabbage is called “Rotkraut” or “Rotkohl” (red kraut or red cabbage), whereas those living in the south, especially Bavarians, call it “Blaukraut.” It's always the same type of cabbage, and the color does not differ, yet those living in the North describe it (and thus presumably perceive it) as red, whereas those in the South perceive it as blue. The garnish is apparently very popular and liked across Germany, thus suggesting that taste (and familiarity) can sometimes beat the mere color property.

### What's the Relationship Between the Resurgence of Blue Food Coloring and Our Increasing Digital Consumption of Food Images?

**Figure 7:**
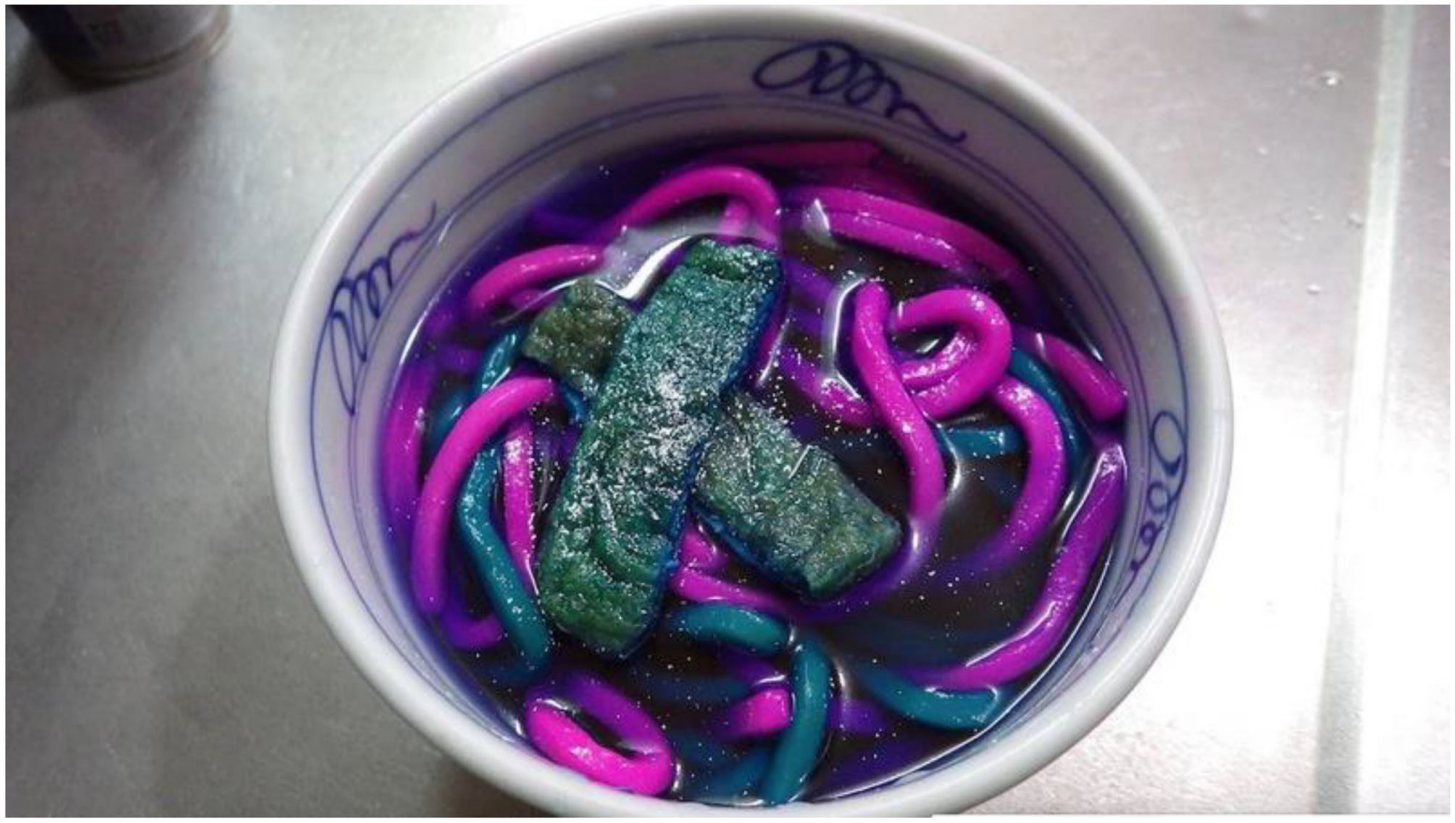
Neon blue noodles created by Kurare. While such visually-captivating dishes generate a lot of interest online. How appealing are such dishes really?

Let me end though with the dishes of biochemist and self-proclaimed “mad scientist” Kurare (Rose, 2015). He has created psychedelic udon noodles using fluorescence chemicals such as new coccine and riboflavin (see Figure 7). While visually stunning, I would argue that they are not appetizing. Ultimately, therefore, one might consider whether the recent resurgence of brightly, one might even say surreally-colored foods would have taken place had it not been for the increasing amount of visual food consumption that occurs by means of our digital technology (Lavis, 2015)? I would also argue that the deconstruction of dishes by modernist/molecular chefs in recent decades has also helped to encourage consumers to think more creatively about the color of the foods they prepare/consume (see Spence and Youssef, 2018; del Moral, 2020).

At the same time, there is also a sense in which people need repeated opportunities to appreciate more challenging material. Carbon and Leder (2005) demonstrated how people may come to appreciate those stimuli that may initially seem relatively uncommon, innovative, and unfamiliar via the Repeated Evaluation Technique. As such, one might wonder whether repeated exposure to blue foods, along with increased elaboration by consumers, might also lead to their increased acceptance in the future. This kind of approach might also lead one to question whether the single-shot evaluation procedure so often used food research in this area might not necessarily be all that useful when it comes to predicting future appreciation.

### What Does Blue Taste Like? Synaesthetic Blue Tastes

Pantone declared Classic Blue (19-4052) its color of the year for 2020. According to Fixsen (2019): “*To augment the 2020 reveal, Pantone included a twist of its own: As part of its marketing campaign, the company partnered with several brands to develop the smell, sound, taste, and texture of Classic Blue. The resulting package included a swatch of suede-like fabric from the Inside, a musk-and-sea-salt-scented candle, a blue, berry-flavored jelly, and a three-minute audio track titled ‘Vivid Nostalgia*^13^.”’ This campaign seems almost synaesthetic in suggesting that blue color has a specific taste/flavor. Potentially relevant here, several synaesthetes have mentioned that they associate certain tastes with a blue-colored concurrent. For example, according to Jaime Smith, a professional sommelier living in Las Vegas, a white wine like Nosiola has a “*beautiful aquamarine, flowy, kind of wavy color to it.”* (quoted in Carlsen, 2013). Meanwhile, the synaesthetic artist Kandinsky also mentioned a synaesthete for whom certain taste inducers give rise to blue color concurrents: “*As far as tasting colors is concerned, many examples are known where this explanation does not apply. A Dresden doctor tells how one of his patients, whom he describes as ‘spiritually, unusually highly developed,’ invariably found that the certain sauce had a ‘blue’ taste, i.e., it affected him like the color blue.”* (Kandinsky, 1977, p. 158; see also Marks, 1978). I would, however, argue that synaesthetic colored-taste concurrents are of little relevance for understanding blue food coloring's relevance to the regular consumer (Spence and Youssef, 2020).

### Blue Plates, Trays, Lights, and Glasses

The appetite-suppressing effects of blue in relation to food apparently extend beyond the food itself. Over the years, it has been suggested that everything from blue plates (see Spence, 2018c, for a review; though see also Schlintl and Schienle, 2020)^14^ to blue trays (Crumpacker, 2006)^15^, and from blue lighting (Cho et al., 2015) to blue dieting spectacles (sold by Yumetai, a Japanese company; Anonymous, 2009) may help to reduce people's food consumption. That said, to date, the evidence supporting such claims would appear to be weak or anecdotal at best. What is more, there are other studies suggesting that high-contrast blue plateware may actually help elderly hospital patients to eat significantly more (not less; Adams, 2013; Spence, 2017b). The latest research suggests that neither blue food nor blue plates necessarily reduces appetite any more than coloring food red, thus confusing matters further (Schlintl and Schienle, 2020). Children have also been reported to consume more, and thus prefer, to eat from colored, rather than white, plates (Brunk and Møller, 2019). Overall, therefore, the evidence supporting the claim that presenting a food against a blue background suppresses appetite currently appears to be rather weak too.

## Conclusions

There is a tension between those commentators/researchers, on the one hand, who want to paint blue as an appetite suppressant (Suzuki et al., 2017), and the food marketers, and Instagrammers who sense the appeal of blue foods (e.g., Hohenadel, 2016; Elgart, 2018), or at least captures visual attention effectively on the shelf or increasingly on the screen (e.g., Garber et al., 2008; Spence, 2016). Is it simply a matter of unusually-colored food being off-putting (e.g., Cardello, 1996; Greenspan, 2009; Ossola, 2016)? Or is there something special about blue, or about unusual color specifically in meat (Vanderbilt, 2015)? As yet, while there is undoubtedly plenty of anecdotal evidence, there is little robust scientific evidence that coloring food blue is any different that coloring food black or purple, say (see Spence, 2020a). If the question is narrowed down, to ask specifically about blue animal protein, the story isn't much clearer/different either.

According to the research summarized here, the associations we hold with, and hence our response to, color in food very much depends on what that food is, and perhaps when historically we are looking at it. Hence, there is likely to be no simple answer to the question of whether blue food is aversive, and if so, how universal that aversion might be. Nevertheless, blue is a special color in the world of food due to its rarity relative to other colors. Rare colors (like blue) capture our attention more effectively in a given context, and at the same time are less likely to have any well-established favor associations. A case can, I think, be made that the recent resurgence of blue foods is intimately linked to our digital consumption of food images.

Ultimately, therefore, there is no single meaning of blue in food. The context, or food format, in which it appears is crucial to determining its meaning. Given the rarity of naturally-blue foods, this means that it is primarily added as a coloring and hence the link between color and flavor is essentially arbitrary (which is not the case for the majority of other food colors). Nevertheless, the majority of consumers nowadays would appear to associate this color in a drink or confectionary product with a sweet fruit flavor (e.g., Shankar M. U. et al., 2010; Velasco et al., 2016). Most often this would appear to be raspberry, of for those who kitsch cocktail loves out there, then the association is with sweet orange due to the popular Blue bols curacao. However, the explosion of new blue drink products in the marketplace in recent years likely means that the flavor associations with this most unusual of food-colors is likely to be more context, format, or glassware dependent than it was, even just a few decades ago.

As for the blue steak story that we started with, that would appear to be nothing more than a much-publicized urban myth. As to why this particular story has proved so popular down through the decades, it might be argued that myths tend to stick when they “sound right,” when they fits our naïve expectations, and/or when we want them to be true (see also Tannenbaum, 2020). Certainly, for those wanting to stress the importance of color to the experience of food and drink then the blue steak example presents what appears to be perhaps the strongest of all support that has been reported to date. As is so often the case, further research will be needed to get to the bottom of the question of whether eating blue meat really does elicit a pronounced aversive reaction in consumers nowadays, as has been so often suggested in the literature in the past.

## Author Contributions

The author confirms being the sole contributor of this work and has approved it for publication.

## Conflict of Interest

The author declares that the research was conducted in the absence of any commercial or financial relationships that could be construed as a potential conflict of interest.

## Footnotes

^1^Wheatley's name seemingly gets more different spellings than Shakespeare: She becomes “Weathley” in Bruno and Pavani (2018, p. 153), and “Wheately” in Thesen et al. (2004), Kappes et al. (2006), and Schlintl and Schienle (2020).

^2^Moir (1936) reported how easily his/her colleagues were fooled by the miscolouring of table jellies and other cakes and confections by mixing-up the typically-occurring colours (e.g., colouring orange or lemon jelly green, and pineapple or apricot jelly red).

^3^The question of off-colour in plant-based imitation meat products has recently come to the fore. According to Tim Geistlinger, vice president of research and development at *Beyond Meat*, one of the leading companies now making plant-based protein products, consumers are not ready for chlorophyll-tinged hamburgers. He was quoted as saying: “*There's no meat that's green unless it's going bad.”* (Vanderbilt, 2015). A British Pathé newsreel from 1959 apparently confirms the displeasing impact that green meat has (see also Tysoe, 1985, p. 13, Tannenbaum, 2020).

^4^I am indebted to Willa Paskin for tracking this down for an episode of *Slate*.

^5^According to Carter (2011): “*Red: to hunter-gatherers, red could denote both ripe fruit and poisonous berries. Today, it's a colour often used to signify sweetness.”*

^6^In terms of packaging, there is also the distinctive blue of Pepsi (Cooper, 1996), and KP Nuts, not to mention the blue sachets that were once included in bags of crisps (Wan et al., 2014b).

^7^Perhaps ironically, in the 19th Century, low-end gin was referred to as ‘blue ruin’ in the UK because of the complexion of those who drank too much of the stuff (Jackson, 2019; see also https://www.victorianlondon.org/entertainment/blueruin.htm).

^8^Woolgar (2018, p. 6) notes how one fifteenth-century cookbook described dishes of food pulps, that is, pottages and broths, as a “blue mush” (blawe mose). An Anglo-Norman culinary collection, from the fourteenth century, also directs the cook that “the colour shall be blue” (Woolgar, 2018, p. 8). Another famous book the *Forme of Cury*, provides instruction on how to make blue food, using a lichen – orchil, that could be red or blue depending on the acid/alkali balance.

^9^In the 19th Century, copper was sometimes added to perk up palid pickled green vegetables (Wilson, 2009; Wollan, 2016, p. 54), while a golden yellow colour was added to margarine to make it look more like butter (Petersen, 1895; Masurovsky, 1939).

^10^According to the traditional Japanese food philosophy *Washoku*, every meal should contain five colours. The term Washoku literally means the harmony of food, a way of thinking about what we eat and how it nourishes us. According to Washoku, the five colors to be included in each meal: black, white, red, yellow, and green (Nobel, 2017).

^11^At the other extreme, of course, some people go for diets of only one colour, or of any colour except white foods (e.g., Rodale, 2017).

^12^Though, it should be remembered that Cortes independently brought potatoes back to Spain, and from there they rapidly spread to other Mediterranean countries (see Tannahill, 1973).

^13^You can listen to the Audio UX track Vivid Nostalgia at https://www.youtube.com/watch?v=SVa6eQ1oRt8. It is, however, important to highlight the distinction between our preference for different colours in a food vs. a non-food context. While blue and green are typically chosen as the favourite colours generally-speaking. In a food context, these two colours are much more likely to appear at the bottom of the list of preferred colours (see Spence, 2019b).

^14^According to the media-friendly synaesthete Jamie Wannerton: “*You serve me food on a blue plate — it just totally messes up the eating sensation.”* (Carlsen, 2013). Though note that it has been suggested that blue plates might increase food consumption in the elderly, many of whom have problems seeing, due to enhanced contrast with typically pale hospital food (e.g., see Adams, 2013).

^15^According to an unsubstantiated anecdote reported by Crumpacker (2006, p. 143): “…*the term blue plate special became popular during the Great Depression because restaurant owners found that diners were satisfied with smaller portions of food if it was served on blue plates*.”
